# Accuracy of Probabilistic Linkage Using the Enhanced Matching System for Public Health and Epidemiological Studies

**DOI:** 10.1371/journal.pone.0136179

**Published:** 2015-08-24

**Authors:** Robert W. Aldridge, Kunju Shaji, Andrew C. Hayward, Ibrahim Abubakar

**Affiliations:** 1 Institute of Health Informatics, University College London, London, United Kingdom; 2 Centre for Infectious Disease Surveillance and Control, Public Health England, London, United Kingdom; 3 Department of Infection & Population Health and MRC Clinical Trials Unit, University College London, London, United Kingdom; FIOCRUZ, BRAZIL

## Abstract

**Background:**

The Enhanced Matching System (EMS) is a probabilistic record linkage program developed by the tuberculosis section at Public Health England to match data for individuals across two datasets. This paper outlines how EMS works and investigates its accuracy for linkage across public health datasets.

**Methods:**

EMS is a configurable Microsoft SQL Server database program. To examine the accuracy of EMS, two public health databases were matched using National Health Service (NHS) numbers as a gold standard unique identifier. Probabilistic linkage was then performed on the same two datasets without inclusion of NHS number. Sensitivity analyses were carried out to examine the effect of varying matching process parameters.

**Results:**

Exact matching using NHS number between two datasets (containing 5931 and 1759 records) identified 1071 matched pairs. EMS probabilistic linkage identified 1068 record pairs. The sensitivity of probabilistic linkage was calculated as 99.5% (95%CI: 98.9, 99.8), specificity 100.0% (95%CI: 99.9, 100.0), positive predictive value 99.8% (95%CI: 99.3, 100.0), and negative predictive value 99.9% (95%CI: 99.8, 100.0). Probabilistic matching was most accurate when including address variables and using the automatically generated threshold for determining links with manual review.

**Conclusion:**

With the establishment of national electronic datasets across health and social care, EMS enables previously unanswerable research questions to be tackled with confidence in the accuracy of the linkage process. In scenarios where a small sample is being matched into a very large database (such as national records of hospital attendance) then, compared to results presented in this analysis, the positive predictive value or sensitivity may drop according to the prevalence of matches between databases. Despite this possible limitation, probabilistic linkage has great potential to be used where exact matching using a common identifier is not possible, including in low-income settings, and for vulnerable groups such as homeless populations, where the absence of unique identifiers and lower data quality has historically hindered the ability to identify individuals across datasets.

## Introduction

The routine collection of electronic health and social care records provides unique opportunities to investigate important research questions in an efficient and powerful way by linking individuals across disparate providers of care. Record linkage has been performed for a number of years in various epidemiological study designs including case control, cohort studies, capture recapture studies and economic evaluations.[1–4]

In a majority of studies, three methods have been used to match records between datasets: Exact matching, deterministic matching, and probabilistic linkage. Exact matching requires records within the two data sets to contain a universally available and unique identifying variable. Many databases across health and social care do not contain such a unique and universally available variable, or accurate and fully available personal identifiable information, limiting the ability to perform exact matching. Deterministic matching can be described as record linkage of two (or more) files based on agreement rules (exact, approximate, and partial) for matching variables. This description of deterministic matching is an updated version of the definition provided by Blakely et al.[5] A recent paper by Bradley et al. provides the following helpful additional description of deterministic matching: " In deterministic matching, the investigator devises a series of steps that will be executed in a particular order to link two datasets. For example, the first step may be to attempt a complete match on SSN (or other unique identifier), sex, and month, day, and year of birth. The second step might be to match on less restrictive criteria, for example, the last four digits of the SSN, sex, and month, day, and year of birth. These steps are continued until as many records as possible are correctly linked between the two datasets".[6] Probabilistic linkage is defined as: “Record linkage of two (or more) files that utilizes the probabilities of agreement and disagreement between a range of matching variables”.[5]

The Enhanced Matching System (EMS) is a probabilistic record linkage program developed to combine data for individuals across two datasets, or within a single dataset for the purposes of de-duplication (de-duplication is not discussed in this manuscript). EMS was developed over several years and can be configured with ease for different matching projects.

EMS was designed and developed by the tuberculosis section at Public Health England using funds from two NIHR grants (RP-PG-0407-10340 and HTA—08\68\01) and builds upon the classic methods described by Newcombe.[7,8] EMS is used operationally by the tuberculosis section at Public Health England for many types of analysis including measuring the levels of drug resistance in tuberculosis cases notified in the UK, and establishing the amount transmission among these cases.[9] Historically, probabilistic linkage has been necessary for this work due to the low recording rates of a unique identifiers between the two datasets (case notifications of tuberculosis to Public Health England and culture positive isolates from tuberculosis reference laboratories across UK) used to establish these estimates. These datasets are probabilistically linked and de-duplicated to form the Enhanced Tuberculosis Surveillance database. In this paper we outline the main features of EMS and present an analysis used to examine its accuracy at matching these two public health tuberculosis datasets.

## Methods

### Enhanced Matching System

EMS is a configurable Microsoft SQL Server database program, currently implemented on Windows 7 and SQL Management studio 2012 and written using the Transact-SQL programming language. The first step in using EMS is data mediation, whereby variables are converted into a pre-specified EMS table structure. To increase the accuracy of the matching, data are then standardised by splitting and parsing of forenames, postcodes, date of birth and addresses, cleaning of country and hospital names when available (for example, through the removal of erroneous spaces at the beginning or end of these country or hospital names), and generating Soundex codes (an algorithm generated index based on the way a name sounds[10]) for name variables. Address information, where available, is split into house or flat number, street name, town or city, postcode and country.

Pairing and blocking is used by EMS to improve the efficiency of the matching process by reducing the number of comparisons required. Depending on available fields within the databases to be matched, blocking involves EMS breaking the records into smaller blocks in three ways: records having the same surname Soundex, year of birth, or first two characters of the postcode area. After all comparisons have been performed within each of the three blocks (or two blocks if either Soundex, year of birth, or postcode area are not available) a cumulative weight is calculated for each comparison pair across the blocks. Duplicate pairs are removed after the blocking by keeping the record pair with the highest weight in a single output table. Three way blocking is used because, for example, if year of birth is missing for one record pair, this record pair will still be linked in one of the other two blocks (surname soundex or postcode district) as long as data are also not missing on these variables in both datasets.

Probabilistic linkage relies on the generation of weights to identify agreements and disagreements between the identifying set of fields in two datasets.[11] EMS generates a weight for matching fields based on the m probability (the probability that the matching variable agrees given that the comparison pair being examined is a match[5]) and the u probability (the probability that a matching variable agrees given that the comparison pair being examined is a non-match[5]). For example, the probability of random agreement for month of birth in two records that are not a true match is approximately 0.08 (or 1/12), which corresponds to the u probability. The m probability depends mainly on the data quality for a matching field. A typical m probability, for an outcome of agreement, is around 0.9 indicating that 90% of matches (for two records are in fact a true match) will have the same value for this matching field. The m probabilities at the start of a matching project are estimates, and several matching runs can be performed within EMS in order to refine values.

Weights are then calculated for each matching field in a pair of records, depending on whether they agree or disagree (Equation 1). The weight for each possible matching pair field is the logarithm to the base 2 (log_2_) of the likelihood ratio. Logarithms to the base 2 are used in probabilistic linkage as per information theory convention.[11]

$$W\;=\;{\text{log}}_{2}\left(\frac{m\;probability}{u\;probability}\right)$$

Equation 1. Formula for calculating individual weights for agreed matches. The m probability and u probability are replaced by (1 –m probability) and (1- u probability) for disagreement matches.

A weight of zero is used when one or both of the fields have missing or unknown values. Where the m probability is greater than the u probability, the weight is positive, and where the 1 –m probability is less than the 1 –u probability then the weight is negative. Therefore agreements are generally positive and disagreements generally negative. EMS carries out matching across multiple fields under the assumption that they are independent. A total weight is obtained for all matching fields in each record pair, as per Equation 2. The logs for individual weights are summed, a process equivalent to multiplying the likelihood ratios.

$$W\;=\;\sum W_{i}$$

Equation 2. Formula for calculating total weight for a record pair

The prior probability of a random pair matching is then calculated (Equation 3[7]) and a threshold is calculated (Equation 4 adapted from the work by Newcombe[7]), above which a record pair is considered matched, using a pre-specified positive predictive value. This threshold is calculated independently of the blocking and is therefore based on all possible pairs, and not the actual number of pairs compared during the matching process.

$$P\left(M\right)\;=\;\frac{m}{n}$$

where

m: estimated number of matches

n: possible matching pairs = (n_1_ ∙ n_2_) / z

n_1_ and n_2_: dataset sizes (obtained directly from the data)

y: number of years

z: correction factor for restrictions on matching, and where z = 1 − *all matches allowed (i*.*e*. *no effect);*

or

$$\frac{y^{2}}{\left(2y-1\right)}\approx \frac{y}{2}–\;matches\;restricted\;to\;within\;1\;year;$$

or

y – matches further restricted to single core year.

Equation 3. The prior probability of a random pair matching

$$Threshold\;=\;{\text{log}}_{2}\left(\frac{P\left(U\right)}{P\left(M\right)}\right)+{\text{log}}_{2}\left(\frac{ppv}{1-ppv}\right)$$

where *P*(*U*): *prior probability of not matching* = 1 − *P*(*M*)

*ppv* = positive predictive value, the probability that record pairs with a total weight above the threshold are truly matches and is specified by the user performing the matching.

Equation 4. Threshold formula

Records below the threshold are considered unmatched and human (manual) review can then be carried out to examine matches close to this threshold (above and below) in order to identify false positives and false negative.

#### Accuracy of EMS

Two datasets were used to examine the accuracy of EMS: case notifications of tuberculosis to Public Health England and a laboratory database of all culture positive isolates from tuberculosis reference laboratories from England, Wales and Northern Ireland. Case notifications are made to Public Health England by healthcare workers looking after patients with tuberculosis and include demographic, and clinical details of cases. The laboratory database contained basic demographic, address information as well as mycobacterial species and drug susceptibility testing results for positive tuberculosis specimens.

All case notifications with a National Health Service (NHS) number for the calendar year 2012 were used for this analysis, along with all de-duplicated (by linking the lab database to itself using EMS) laboratory database records with an NHS number for the period 1^st^ October 2011–31^st^ March 2013. Laboratory database records for the three months before and after the calendar year of 2012 were included as tuberculosis cases may be notified before or after the microbiological result. This strategy therefore aims to increase the number of possible matches across the two datasets.

Exact matching using NHS number was used as a gold standard to identify linked records. NHS number is a ten digit unique identifier for a patient and is used throughout the health service. It is often formatted 3-3-4, with separating space or hyphen (e.g. 123-456-7890). NHS numbers included in the final analysis as the gold standard were checked for validity. Simple descriptive analysis was performed to examine differences between tuberculosis notification and laboratory isolate records with and without NHS numbers.

After exact matching, case notifications and the laboratory database were probabilistically linked by EMS using first name, surname, date of birth, sex, address details (including postcode) data, first name soundex and surname soundex. NHS number was not included as a matching variable in the probabilistic linkage in order that the matching was independent of this gold standard identifier. Three blocking passes were performed with surname soundex, year of birth, and the first two characters of the postcode respectively. A descriptive analysis stratified by NHS number availability and validity was performed to examine missing data on variables used for the linkage from the laboratory, case notifications and an example pre-entry screening dataset. All records except for those with a missing or invalid NHS number were included in the accuracy analysis.

The matching threshold was calculated using a value of y = 1 and ppv = 0.99. A decision was taken a-priori to manually review records with a matching score of 10 above and below the matching threshold. One author (RWA) performed the manual review and was not blinded to the analysis, but did not have information on NHS number for record pairs when performing this manual review. As probabilistic linkage was performed without including NHS number as matching variable, manual review included all records regardless of whether or not they were exact matched by NHS number. Outcomes used to assess accuracy of the matching process were sensitivity, specificity, positive and negative predictive values. A full description of how these were calculated is provided in S1 Table. Exact confidence intervals (to the 95% level) were calculated in Stata version 13 using a binomial distribution.

Four sensitivity analyses were carried out. Firstly, probabilistic linkage was performed using first name, surname, date of birth, sex, address details (including postcode), first name soundex, surname soundex but without manual review. Secondly, probabilistic linkage was performed without address variables and without manual review. Thirdly, to examine the effect that a larger proportion of non-English names would have on the accuracy, matching was performed without address variables and manual review, but in a case notifications dataset that only included non-UK born individuals. Fourthly, a sensitivity analysis was carried out to determine the impact of varying the automatically calculated weight threshold on outcome measures without manual review, but including all matching variables except NHS number.

Ethical approval was not required for this accuracy analysis, as Public Health England has Health Research Authority approval to hold and analyse national surveillance data for public health purposes under Section 251 of the NHS Act 2006.

## Results

A total of 8,751 records were extracted from the case notifications dataset and 7,538 unique records from the laboratory database. 67.8% of case notifications and 23.3% of the laboratory database records contained valid NHS numbers. Comparing the characteristics of records with and without valid NHS number showed differences in age and higher rates of Isoniazid resistance in those records without an NHS number (Table 1). No other differences were found.

**Table 1:** Descriptive analysis of laboratory dataset for records with and without an NHS number.

		NHS Number				
		Available and valid		Not available or invalid		
	All	N	%	N	%	p-value*
**All**	7538	1759	23.3	5779	76.7	
**Age group in years**						
0 to 14	122	40	32.8	82	67.2	
15 to 44	4724	990	21.0	3734	79.0	
45 to 64	1576	409	26.0	1167	74.0	
65 and over	1061	320	30.2	741	69.8	<0.001
Missing**	55	0	0	55	100.0	
**Sex of case**						
Female	2941	726	24.7	2215	75.3	
Male	4355	1012	23.2	3343	76.8	
Missing	242	21	8.7	221	91.3	0.15
**Isoniazid sensitivity result**						
Resistant	508	95	18.7	413	81.3	
Sensitive	6801	1629	24.0	5172	76.0	
Missing	229	35	15.3	194	84.7	0.007
**Ethambutol sensitivity result**						
Resistant	84	19	22.6	65	77.4	
Sensitive	7217	1699	23.5	5518	76.5	
Missing	237	41	17.3	196	82.7	0.84
**Rifampicin sensitivity result**						
Resistant	142	29	20.4	113	79.6	
Sensitive	7181	1697	23.6	5484	76.4	
Missing	215	33	15.3	182	84.7	0.37
**Pyrazinamide sensitivity result**						
Resistant	108	26	24.1	82	75.9	
Sensitive	7161	1690	23.6	5471	76.4	
Missing	269	43	16.0	226	84.0	0.91

*Chi squared test, not including missing data for each variable other than NHS number

**It was not possible to calculate the exact age for these records as the date of their laboratory result was not recorded, but date of birth was available for all records.

A greater number of differences were seen between those records with and without a valid NHS number in the case notifications dataset. All variables apart from those for drug sensitivity testing and site of tuberculosis disease showed a difference between those with and without an NHS number (Table 2). These data show that women, ethnic minority groups, individuals not born in the UK, and individuals with at least one social risk factor for tuberculosis (including drug use, homelessness, alcohol misuse/ abuse, prison) were more likely not to have an NHS number.

**Table 2:** Descriptive analysis of case notifications dataset for records with and without an NHS number.

		NHS Number				
		Available and valid		Not available or invalid		
	All	N	%	N	%	p-value*
Total	8751	5931	67.8	2820	32.2	
**Age**						
<14	414	277	66.9	137	33.1	
15–44	5291	3495	66.1	1796	33.9	
45–64	1830	1273	69.6	557	30.4	
65+	1216	886	72.9	330	27.1	<0.001
**Sex**						
Female	3706	2619	70.7	1087	29.3	
Male	5045	3312	65.6	1733	34.4	<0.001
**Ethnic group**						
White	1814	1316	72.5	498	27.5	
Black-Caribbean	175	121	69.1	54	30.9	
Black-African	1358	859	63.3	499	36.7	
Black-other	71	41	57.7	30	42.3	
Indian	2295	1471	64.1	824	35.9	
Pakistani	1418	1098	77.4	320	22.6	
Bangladeshi	320	194	60.6	126	39.4	
Chinese	95	67	70.5	28	29.5	
Mixed/other	979	625	63.8	354	36.2	
Missing	226	139	61.5	87	38.5	<0.001
**UK Born**						
No	6125	4049	66.1	2076	33.9	
Yes	2256	1652	73.2	604	26.8	
Missing	370	230	62.2	140	37.8	<0.001
**Site of disease**						
Extra-pulmonary disease only	4095	2754	67.3	1341	32.7	
Pulmonary, with or without extra-pulmonary disease	4563	3128	68.6	1435	31.4	
Missing	93	49	52.7	44	47.3	0.20
**Social risk factor** **						
No	7683	5210	67.8	2473	32.2	
Yes	637	390	61.2	247	38.8	
Missing	431	331	76.7	100	23.3	<0.001
**Isoniazid sensitivity result**						
Sensitive	4801	3206	66.8	1595	33.2	
Resistant	351	235	67	116	33	
Missing	3599	2490	69.2	1109	30.8	0.95
**Ethambutol sensitivity result**						
Sensitive	5087	3396	66.8	1691	33.2	
Resistant	51	35	68.6	16	31.4	
Missing	3613	2500	69.2	1113	30.8	0.78
**Rifampicin sensitivity result**						
Sensitive	5060	3377	66.7	1683	33.3	
Resistant	91	63	69.2	28	30.8	
Missing	3600	2491	69.2	1109	30.8	0.62
**Pyrazinamide sensitivity result**						
Sensitive	5043	3364	66.7	1679	33.3	
Resistant	45	33	73.3	12	26.7	
Missing	3663	2534	69.2	1129	30.8	0.35

*Chi squared test, not including missing data for each variable other than NHS number

**At least one social risk factor including drug use, homelessness, alcohol misuse/ abuse, prison

**Fig 1:**
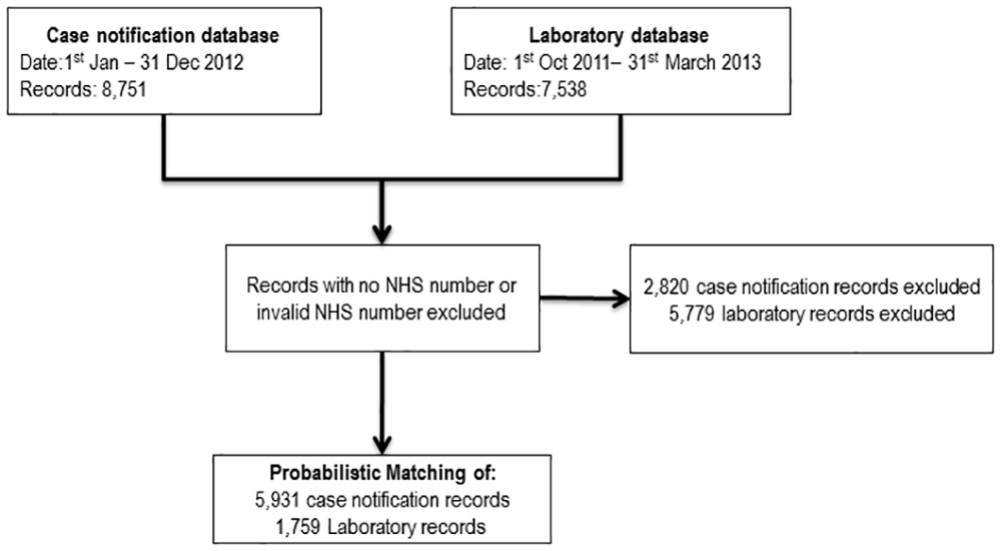
Flow chart of datasets used for study.

For assessment of accuracy, only records with an NHS number were included in the probabilistic linkage. The final probabilistic linkage dataset therefore consisted of 5,931 records from the case notifications database and 1,759 records from the laboratory database (Fig 1). In this final dataset used for the assessment of accuracy (those with an available and valid NHS number) there was one record in the case notification dataset missing surname and date of birth, but not first name (Table 3). One record in the laboratory dataset was missing first name and surname information, and no records were missing date of birth. 13 (0.2%) records were missing the first line of their address in the case notifications database and 232 (15.1%) in the laboratory database. 6 (0.1%) records were missing postcode information in the case notifications database, and 126 (7.2%) were missing in the laboratory dataset. 21 (1.1%) records were missing information on sex in the laboratory database, and none in the case notifications. There were low amounts of missing data for first name, surname, date of birth and sex for those records with a missing or invalid NHS number. With the exception of the address line 2 (typically city, town, or local area) in the case notifications dataset, records with a missing or invalid NHS number had higher levels of missing data than those with a valid and available NHS number. The example dataset from a pre-entry screened migrant population shows the high quality data on linkage variables that is available internationally for important public health analyses.

**Table 3:** Description of missing data on variables used for the linkage from the laboratory, case notifications and an example pre-entry screening dataset, by NHS number availability and validity.

	**Missing data for linkage variables**			
	**NHS number available and valid**		**NHS number not available or invalid**	
	**N**	**%**	**N**	**%**
**Laboratory dataset**				
All	1759	100%	5779	100%
Firstname	1	0%	13	0%
Surname	1	0%	0	0%
Date of birth	0	0%	0	0%
Sex	21	1%	173	3%
Address line 1*	232	13%	4513	78%
Address line 2**	1023	58%	5387	93%
Postcode	126	7%	3939	68%
**Case notifications dataset**				
All	5931	100%	2820	100%
First name	0	0%	0	0%
Surname	1	0%	0	0%
Date of birth	1	0%	1	0%
Sex	0	0%	0	0%
Address line 1*	13	0%	8	0%
Address line 2**	2918	49%	1302	46%
Postcode	6	0%	9	0%
**Pre-entry screening dataset**				
All			640808	100%
Firstname	-	-	13078	2%
Surname	-	-	4824	1%
Date of birth	-	-	910	0%
Sex	-	-	786	0%
Nationality	-	-	796	0%

*E.g. house number and street name

**E.g. city.

Exact matching between the two datasets using NHS number identified 1,071 matched pairs. In the case notifications database 4,860 (81.9%) records had no matching pair in the laboratory database, and 688 (39.1%) records from the laboratory database had no matching pair in the case notifications database.

Probabilistic linkage of the case notifications database to the laboratory database identified 1088 linked pairs using the EMS generated threshold of 19.98. Manual review of 67 pairs with a weight between 10 and 30 resulted in two below the threshold being changed to matches, and three results above the threshold being marked as not matching. A total of 19 records that represented multiple matches (for example, one record in the lab dataset that matches with two non-duplicate records in the case notifications dataset, or duplicate matches created when combining results from the three blocking passes) were removed without manual review. The pair with the highest weight was chosen as the final match to include in the analysis. A total of 1068 matches were therefore identified by the EMS process after manual review and de-duplication.

### Accuracy of probabilistic linkage

Using the threshold of 19.98, and after manual review of records above and below the threshold and de-duplication of matches, 1,066 records were identified as true positives and 5,546 as true negatives (Table 4). At the threshold of 19.98, and with manual review, there were 2 false positives and 5 false negatives. The false negatives had the same date of birth, and several cases had first names and surnames that were switched, one sex was unknown, and all had different addresses. Both false positives had the same first name, surname, date of birth, sex and address, but different NHS numbers suggesting an error in the recording of NHS number.

**Table 4:** Comparison of matches identified by exact linkage using NHS number, and the probabilistic linkage process (without NHS number) and with de-duplication and manual review.

		Exact matching (NHS Number)		
		+ve	-ve	Total
Probabilistic (EMS)	+ve	1066	2	1068
	-ve	5	5546	5551
	Total	1071	5548	6619

Note: the total denominator is calculated using the number of exactly linked pairs (1071), plus the 4,860 records in the case notifications database had no matching pair in the laboratory database, and 688 records from the laboratory database had no matching pair in the case notifications database (i.e. 1071 + 4860 + 688 = 6619).

The sensitivity of the probabilistic linkage was 99.5% (95%CI: 98.9, 99.8) and specificity 100.0% (95%CI: 99.9, 100.0). The corresponding positive predictive value was 99.8% (95%CI: 99.3, 100.0) and negative predictive value 99.9% (95%CI: 99.8, 100.0).

A series of sensitivity analyses were carried out to examine the performance of the linkage compared to a gold standard using different assumptions (Table 5). Without manual review, but using all linkage variables except NHS number, sensitivity was 99.3% (95%CI: 98.7, 99.7) and specificity 99.9%(95%CI: 99.8, 100.0). Matching without NHS number, address variables and manual review resulted in a sensitivity of 97.1% (95%CI: 95.9, 98.0) and specificity 100.0%(95%CI: 99.9, 100.0). To examine the effect of having a larger proportion of non-English names has on accuracy, matching was performed without address variables and manual review in a case notifications dataset with only non-UK born individuals and found a sensitivity of 96.5% (95%CI: 94.9, 97.8) and specificity 100.0% (95%CI: 99.8, 100.0).

**Table 5:** Calculation of sensitivity and specificity for probabilistic matching without manual review, not including address variables and using an ETS dataset that only including non-UK born individuals.

Variables used for matching	Sensitivity	Specificity
All with manual review	99.5% (95%CI: 98.9, 99.8)	100.0% (95%CI: 99.9, 100.0)
All without manual review	99.3% (95%CI: 98.7, 99.7)	99.9%(95%CI: 99.8, 100.0)
No address variables (without manual review)	97.1% (95%CI: 95.9, 98.0)	100.0%(95%CI: 99.9, 100.0)
No address variables, only individuals born outside UK (without manual review)	96.5% (95%CI: 94.9, 97.8)	100.0% (95%CI: 99.8, 100.0)

Varying the threshold between a credible range of 10 and 50 resulted in sensitivity changing from 86.1% (95%CI: 83.9, 88.1) to 99.6%(95%CI: 99.0, 99.9; Table 6). In this same analysis, specificity ranged from 99.5% (95%CI: 99.3, 99.7) to 100.0% (95%CI: 99.8, 100.0).

**Table 6:** Estimated sensitivity, specificity, positive predictive value and negative predictive values when varying the thresholds used to determine matched pairs. Manual review not performed.

Threshold weight score used	True positives	Probabilistic matches	True negatives	Sensitivity	Specificity	Positive predictive value	Negative predictive value
10	1067	1094	5521	99.6%	99.5%	97.5%	99.9%
15	1065	1086	5527	99.4%	99.6%	98.1%	99.9%
20	1064	1069	5543	99.3%	99.9%	99.5%	99.9%
25	1060	1062	5546	99.0%	100.0%	99.8%	99.8%
30	1047	1049	5546	97.8%	100.0%	99.8%	99.6%
35	1022	1024	5546	95.4%	100.0%	99.8%	99.1%
40	987	989	5546	92.2%	100.0%	99.8%	98.5%
45	946	948	5546	88.3%	100.0%	99.8%	97.8%
50	922	924	5546	86.1%	100.0%	99.8%	97.4%

**Fig 2:**
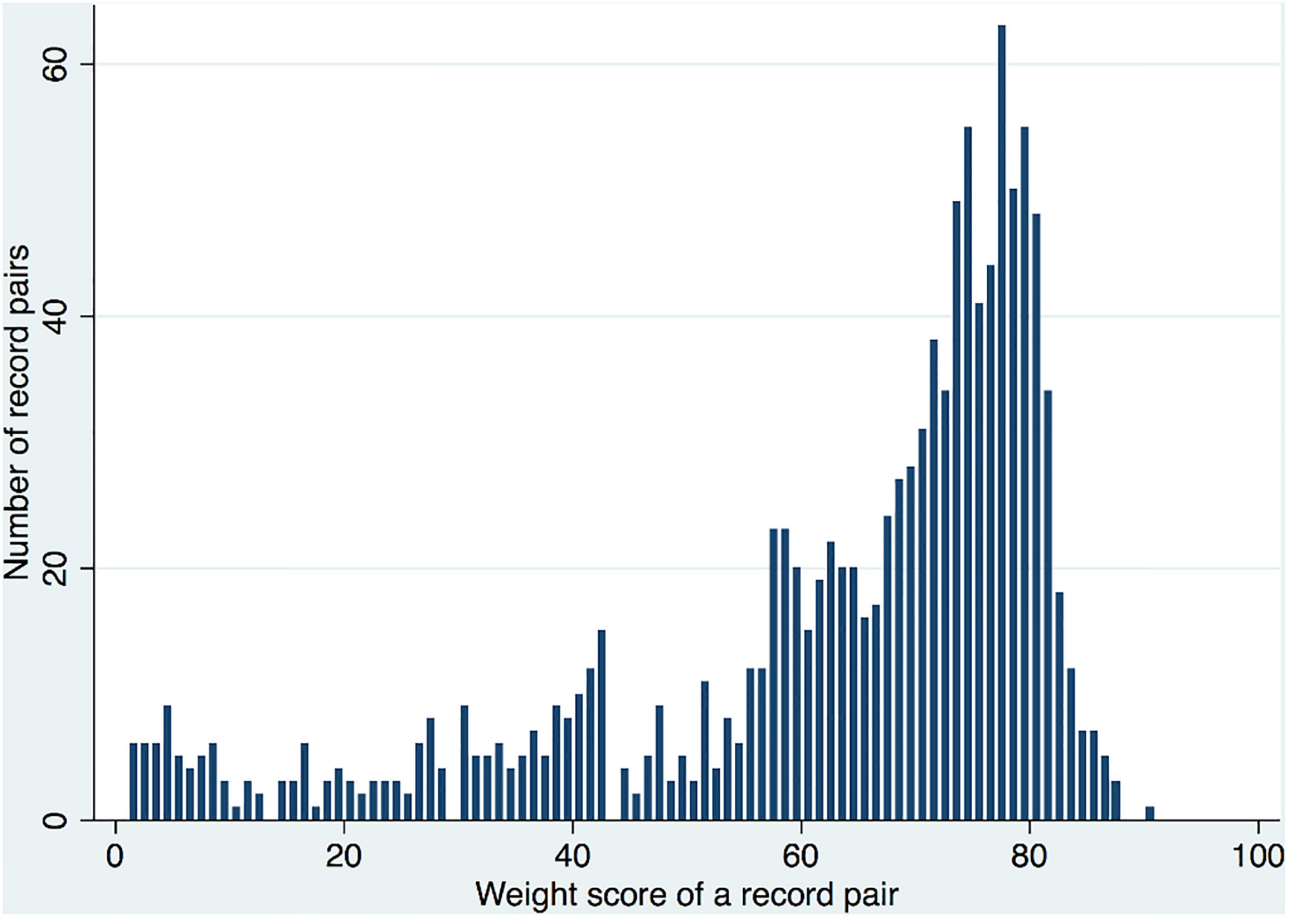
Number of pairs by total weight score, without manual review or de-duplication and not including NHS number. Only pairs with a total weight score greater than zero are presented.

The distribution of the weights for the matching process is shown in Fig 2, which presents pairs with a total weight score greater than zero and therefore excludes the very large number of non-matches. The number of matches increased rapidly after a weight of around 50 and decreasing rapidly after the mode of 77.

## Discussion

The Enhanced Matching System uses probabilistic linkage and had high accuracy when compared to a gold standard exact matching based upon NHS number. Probabilistic linkage was most accurate when including address variables and using the automatically generated threshold for determining matches with manual review. Accuracy remained relatively high even after exclusion of address information from the linkage process, in linkage without manual review, and in a case notifications dataset that only included non-UK born individuals. Varying the weight threshold used to determine matches affected the sensitivity and specificity of probabilistic linkage.

The characteristics of records in the case notification and laboratory datasets with missing or invalid NHS numbers were examined. As a result of the lack of demographic data in the laboratory database, the only differences found were for age and Isoniazid resistance, which has been associated with an outbreak of tuberculosis in homeless, prison, certain ethnic groups and drug using populations in London.[12] In the case notifications database there were more differences for records with and without NHS numbers. NHS numbers were missing for more records in those aged between 15 and 44, males, ethnic minorities, non-UK born and individuals with social risk factors. It is not surprising that there were greater levels of missing NHS numbers for those with social risk factors, as these individuals tend to have poorer access and usage of NHS services.[13]

The dataset used for main analysis, which only included those records with a valid NHS number, comprised of low levels of missing data on linkage variables and is therefore likely to represent the higher end of accuracy achievable by EMS. The datasets used in this study contained a high proportion of individuals not born in the UK, and ethnic minority groups making this analysis relevant to these populations. The sensitivity analysis examining probabilistic linkage in a case notifications dataset that only contained UK born individuals examined this issue further, and found that the accuracy of EMS remained high in this analysis.

NHS number is a unique identifying variable that is verifiable and reliable, making it an ideal gold standard comparator. However, it is not always available in public health datasets, demonstrated by the fact that it was available and valid for only 23.3% of laboratory database records, and 67.8% of case notification records. The high level of missing or invalid NHS numbers highlights a strength of probabilistic linkage over exact matching, in addition to the fact that it is able to account for errors and omissions of other data on linkage variables. However, the accuracy of probabilistic matching is still dependent on what identifying variables are available, and the quality of data contained within these variables. The amount of missing data on linkage variables (e.g. first name, surname, date of birth, sex and address) for those records with or without a valid NHS number was similar in the case notifications dataset, but the laboratory dataset had more missing data in those records without an NHS number. This may mean that linkage performs less well on such records, but this issue is complicated by the fact that the laboratory dataset may contain records that should not have an NHS number or address information (e.g. those isolated from animals). It is therefore difficult to state with certainty, whether linkage to the laboratory records without a valid NHS number will be lower or not compared to those with a valid NHS number.

The example dataset from a pre-entry screened migrant population presented in table 3 demonstrates the high quality of international data that can be used for public health analyses. A linkage between this pre-entry screened population and the case notifications dataset has enabled an investigation into the risk factors associated with being notified as a case of tuberculosis in migrants to the UK and has informed the development of policy at Public Health England.

Not all tuberculosis cases are microbiologically confirmed, and it is therefore not unexpected that the case notification dataset included more individuals than the laboratory database. Conversely it is possible that some patients recorded in the laboratory database were not notified as a case of tuberculosis and are therefore not included in the case notifications dataset. There are many reasons for laboratory records not to be found in the case notification dataset including those isolated from animals, cases reported to the case notification surveillance system in subsequent years after laboratory confirmation, cases of non-tuberculosis mycobacterium, samples positive due to laboratory contamination, and samples originating from the Channel Islands which are not notified to the surveillance system.

The accuracy of matching was assessed using a relatively small dataset. Repeating the analysis on a larger dataset may have some affect on the measures of accuracy found in this analysis, depending on the frequency of matches between the datasets. For this analysis, matching was performed between two data sets of the same disease, using several matching variables including names, date of birth and address data that had low levels of missing data, and therefore a high degree of matching is to be expected. In scenarios where a small sample is being matched into a very large database (such as national records of hospital attendance) then the positive predictive value or sensitivity will drop according to the prevalence of the sample within the larger database. This may lead to overestimation of the frequency of occurrence of the sample within the larger database (i.e. identifying more matches in the larger database than there truly were) If this linked dataset was used to calculate incidence or prevalence of an outcome, such a bias within the linked dataset would result in estimates were higher than the true values.[5]

Manual review of matches introduces subjectivity into the matching process and this may have implications for repeatability of this part of the linkage. Additionally, as the treating tuberculosis clinician does not perform the manual review (as is usually the case), those performing manual review were quite removed from the clinical situation and this may bias results. Such human error is likely to be differentially (and not randomly) biased. Further work should be carried out to assess the impact of the subjectivity of the manual review process, and examine the applicability of developing rules to provide consistent and potentially unbiased results.

When used for epidemiological studies, errors in probabilistic linkage have the potential to impact on findings and conclusions drawn. Linkage is typically used for the generation of outcome data in cohort studies, for example, to determine the vital status for individual participants by matching data into death registries. Assuming there is non-differential misclassification bias of exposure variables, false positive links will bias risk ratios and risk differences towards the null.[5,14,15] Risk ratios will be unaffected by false negative results (assuming there is non-differential misclassification bias), however, risk differences in cohort studies will be biased towards the null. False positive and negative probabilistic links in public health surveillance or outbreak studies will also result in under or over estimation of the number of cases. We are not aware of studies that have analysed this directly, but capture recapture studies attempt to examine this issue and in the UK have demonstrated the utility of the probabilistic linkage for improving data quality and case ascertainment levels.[16]

For this study, there were insufficient numbers of false positive and false negative results to enable examination of the issue of misclassification bias further, however, the fact that there was very high sensitivity and specificity means misclassification should introduce minimal bias. Further research is needed to understand the implications of these misclassification biases, particularly when such analyses are being conducted to estimate disease incidence or prevalence.

Probabilistic linkage has been widely adopted in research and service public health analyses. Several studies have previously examined the accuracy of probabilistic linkage using datasets ranging in size from 250 to 3,131,176 records. [17] Findings in these studies are consistent with the results presented in this analysis, with sensitivities ranging from 86% (database sizes: 250 records with N of second dataset not published [18]) to 99.2% (database sizes: 6,000 records in both[19]), and specificity ranging from 99.4% (database sizes: 6,000 records in both[19]) to 100% (database sizes: 822 and 450[20]). Variation in these results may be due to algorithms used for probabilistic linkage, as well as characteristics of the datasets such as the rates of missing data, errors and omissions which impact on the results.

## Conclusions

The Enhanced Matching System has been found to have high accuracy for the probabilistic linkage of public health datasets. With the establishment of national electronic datasets across health and social care, the accuracy of this software enables previously unanswerable research questions to be tackled.[21–23] Probabilistic linkage has great potential to be used where exact matching using a common identifier is not possible, including in low-income settings, and for vulnerable populations such as homeless populations. In these situations, the absence of unique identifiers has historically hindered the ability to identify individuals across separate datasets in order to establish outcomes or exposures as required by many types of epidemiological study design.

## Supporting Information

S1 Table Description of how sensitivity, specificity, positive and negative predictive values were calculated. (DOCX)
