# Supplementary material for: Accuracy of Probabilistic Linkage Using the Enhanced Matching System for Public Health and Epidemiological Studies
Source: PLoS One. 2015 Aug 24;10(8):e0136179. doi: 10.1371/journal.pone.0136179 (PMC4547731; doi:10.1371/journal.pone.0136179)
Supplement: S1 Table — (DOCX) [file pone.0136179.s001.docx]

**S1 Table**

Description of how sensitivity, specificity, positive and negative predictive values were calculated.

| Probabilistic (EMS) |  | Deterministic (NHS Number) | | |
| --- | --- | --- | --- | --- |
|  |  | +ve | -ve | Total |
|  | +ve | a | b | a+b |
|  | -ve | c | d | c+d |
|  | Total | a+c | b+d | a+b+c+d |

Sensitivity = a/a+c

Specificity = d/b+d

Positive predictive value= a/a+b

Negative predictive value = d/(c+d)
